# Identification of a potent and selective LAPTc inhibitor by RapidFire-Mass Spectrometry, with antichagasic activity

**DOI:** 10.1371/journal.pntd.0011956

**Published:** 2024-02-15

**Authors:** Maikel Izquierdo, De Lin, Sandra O’Neill, Lauren A. Webster, Christy Paterson, John Thomas, Mirtha Elisa Aguado, Enrique Colina Araújo, Daniel Alpízar-Pedraza, Halimatu Joji, Lorna MacLean, Anthony Hope, David W. Gray, Martin Zoltner, Mark C. Field, Jorge González-Bacerio, Manu De Rycker

**Affiliations:** 1 Centre for Protein Studies, Faculty of Biology, University of Havana, La Habana, Cuba; 2 Drug Discovery Unit, Wellcome Centre for Anti-Infectives Research, University of Dundee, Dundee, United Kingdom; 3 Department of Biochemistry, Faculty of Biology, University of Havana, La Habana, Cuba; 4 Centre for Pharmaceuticals Research and Development, La Habana, Cuba; 5 Wellcome Centre for Anti-Infectives Research, Division of Biological Chemistry and Drug Discovery, School of Life Sciences, University of Dundee, Dundee, United Kingdom; 6 Department of Parasitology, Faculty of Science, Charles University in Prague, Biocev, Vestec, Czech Republic; 7 School of Life Sciences, University of Dundee, Dundee, United Kingdom; 8 Biology Centre, Czech Academy of Sciences, Institute of Parasitology, České Budějovice, Czech Republic; University of Texas at El Paso, UNITED STATES

## Abstract

**Background:**

Chagas disease is caused by the protozoan parasite *Trypanosoma cruzi* and leads to ~10,000 deaths each year. Nifurtimox and benznidazole are the only two drugs available but have significant adverse effects and limited efficacy. New chemotherapeutic agents are urgently required. Here we identified inhibitors of the acidic M17 leucyl-aminopeptidase from *T*. *cruzi* (LAPTc) that show promise as novel starting points for Chagas disease drug discovery.

**Methodology/Principal findings:**

A RapidFire-MS screen with a protease-focused compound library identified novel LAPTc inhibitors. Twenty-eight hits were progressed to the dose-response studies, from which 12 molecules inhibited LAPTc with IC_50_ < 34 μM. Of these, compound 4 was the most potent hit and mode of inhibition studies indicate that compound 4 is a competitive LAPTc inhibitor, with K_i_ 0.27 μM. Compound 4 is selective with respect to human LAP3, showing a selectivity index of >500. Compound 4 exhibited sub-micromolar activity against intracellular *T*. *cruzi* amastigotes, and while the selectivity-window against the host cells was narrow, no toxicity was observed for un-infected HepG2 cells. *In silico* modelling of the LAPTc-compound 4 interaction is consistent with the competitive mode of inhibition. Molecular dynamics simulations reproduce the experimental binding strength (-8.95 kcal/mol), and indicate a binding mode based mainly on hydrophobic interactions with active site residues without metal cation coordination.

**Conclusions/Significance:**

Our data indicates that these new LAPTc inhibitors should be considered for further development as antiparasitic agents for the treatment of Chagas disease.

## Author summary

*Trypanosoma cruzi* is a single cell eukaryotic parasite that infects humans and animals. Infection can result in Chagas disease, a debilitating chronic disease that frequently affects the heart. The major downside of the current treatments is their side-effects, which frequently prevent patients from completing their treatment course. New, safer medicines are therefore urgently needed. Here we sought to enable the development of new medicines by identifying inhibitors of a key parasite enzyme; acidic M17 leucyl-aminopeptidase (LAPTc). We identified 28 LAPTc inhibitors, among more than 3000 compounds tested. The most potent inhibitor, compound 4, inhibited parasite growth and did not inhibit the equivalent human aminopeptidase. Using computational tools, we predicted how this compound binds to the enzyme. Our results indicate that the LAPTc inhibitors could be considered for further development as antiparasitic agents for the treatment of Chagas disease.

## Introduction

Chagas disease is a neglected tropical disease caused by the protozoan kinetoplastid parasite *Trypanosoma cruzi*, affecting mainly Latin America, but also present in migrant populations in North America, Europe, Japan and Australia [1]. Approximately 6–7 million people are currently infected [2], with ~50,000 new cases and ~10,000 deaths annually. The parasite is transmitted between humans and local fauna by hematophagous triatomine insects [3].

Infection progresses through three phases: acute, indeterminate and chronic [4,5]. The acute phase (4–8 weeks) is characterized by the presence of *T*. *cruzi* in blood and patients are usually asymptomatic or have non-specific symptoms of infection (fever, anorexia, malaise, lymphadenopathy, etc). The indeterminate phase is typified as silent, with no overt symptoms [4,5], but 20–30% of infected people progress to the symptomatic chronic phase, manifested as cardiomyopathy, neuropathy or gastrointestinal disorders [6,7].

Only two drugs are currently available: nifurtimox and benznidazole, but these nitroheterocyclic compounds are poorly tolerated and require protracted treatment regimens [8]. New and effective chemotherapies are urgently required.

For infectious diseases, rational drug discovery is frequently based on the identification, characterization and validation of molecular targets in the disease-causing agent. A key advantage of this strategy is to facilitate structure-guided compound design and rapid progress in drug discovery [9]. Proteases have been successfully targeted in many diseases [10], including infectious diseases [11] where they play key roles in microbial physiology [12,13].

All life-cycle stages of *T*. *cruzi* express an acidic M17 metallo-aminopeptidase (LAPTc), responsible for the main leucyl aminopeptidase (LAP) activity in parasite extracts [14]. Although LAPTc has not been validated as a target, it may be involved in nutrient supply, since the parasite lacks the biosynthetic pathway for leucine biosynthesis [14,15]. In agreement with the critical functions proposed, the LAPTc inhibitor arphamenine A [16] inhibits *in vitro* growth of *T*. *brucei brucei*, a parasite closely related to *T*. *cruzi* [17]. In addition, TbLAP1, an M17 LAP from *T*. *brucei*, participates in kinetoplast DNA segregation and silencing causes a delay in cytokinesis [18]. Interestingly, the classical metalo-aminopeptidase inhibitor bestatin [19] inhibits LAPTc in epimastigotes, the insect parasite stage [20]. Therefore, LAPTc inhibition by bestatin-like molecules is a potential strategy to inhibit parasite growth and for development of anti-chagasic drugs.

M17 LAPs could also be exploited as targets in other parasites. The M17 LAP from *Plasmodium falciparum* (PfA-M17) is essential as it is involved in haemoglobin digestion [21] and other housekeeping functions, as suggested by results obtained with a specific bestatin-derived inhibitor [22]. Knockdown of M17 LAP in the parasite *Acanthamoeba castellanii*, or treatment with EDTA, 1,10-phenanthroline (metallo-protease inhibitors) or bestatin, lead to cell wall changes, closely related to inhibition of encystation [23]. Bestatin inhibition of *Babesia bovis* growth has been attributed to inhibition of M17 LAP [24]. Two M17 LAPs from *Schistosoma mansoni* could be involved in haemoglobin degradation, surface membrane remodelling and egg hatching, as suggested by RNAi-mediated knockdown or treatment with bestatin [25]. Finally, knockout of *Toxoplasma gondii* M17 LAP inhibits the ability to invade cells in culture, reduces replication and attenuates virulence in mice [26]. Therefore, these enzymes may be important for developing therapeutic strategies for many parasitic diseases.

**Fig 1:**
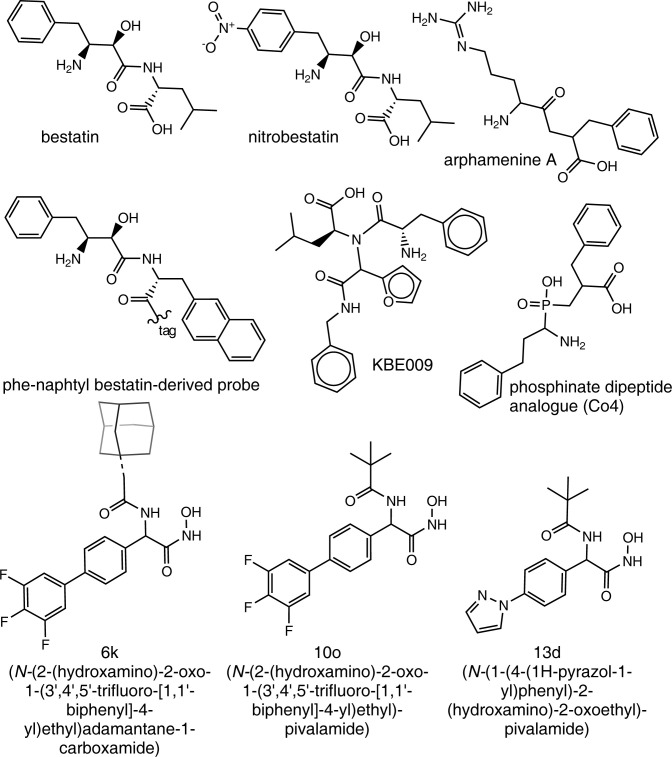
Structures of previously described M17 LAP inhibitors [14,16,21,22,27–31].

M17 LAP inhibitors are dipeptide-like compounds, with hydrophobic and bulky substituents. Only a few LAPTc inhibitors have been identified; bestatin [14], the bestatin-based peptidomimetic KBE009 [27] and arphamenine A [16] (Fig 1). PfA-M17 inhibitors have also been reported. For example, bestatin (inhibition constant (K_i_) = 25 nM [28]; Fig 1) and nitrobestatin (K_i_ = 2.7 nM [21]; Fig 1). Both compounds have isobutyl and benzyl substituents (*p*-nitro-benzyl for nitrobestatin). In addition, PfA-M17 is inhibited by the bestatin-derived activity-based probe Phe-Naphtyl (K_i_ = 29 nM [22]; Fig 1). The phosphinate dipeptide analogue Co4, with two phenyl rings, inhibits also PfA-M17 (K_i_ = 13 nM [28]; Fig 1). Finally, PfA-M17 is inhibited by the hydroxamates 13d (K_i_ = 28 nM; has pyrazole, phenyl and terbutyl groups [29]; Fig 1), 10o (K_i_ = 60 nM; two phenyl rings and the terbutyl group [30]; Fig 1) and 6k (K_i_ = 28.9 nM; three hydrophobic and bulky rings in its structure [31]; Fig 1).

Here, we identified a potent LAPTc inhibitor through screening a small protease-focused compound collection, using RapidFire-MS. The inhibitor is competitive for the LAPTc substrate, selective with respect to human LAP3 and has sub-micromolar potency against intracellular *T*. *cruzi* amastigotes. Molecular docking reveals a binding mode consistent with competitive inhibition. Molecular dynamics simulations reproduce the experimental binding strength (-8.95 kcal/mol) and indicate that binding is mainly driven by hydrophobic interactions rather than metal cation coordination. This molecule represents a valuable starting point for development of a new antichagasic drug.

## Methods

### Compound library

The protease library screened contained 3,329 compounds that harbour common protease inhibitor motifs. Compounds were selected and acquired from commercial sources. All compound structures are available in ChEMBL (see Data Availability statement).

### Recombinant LAPTc production

Recombinant LAPTc production was described in [32]. Briefly, a LAPTc construct was designed, codon optimized for expression in *Escherichia coli*, synthesized and cloned into the vector pET-19b (Eurofins Genomics, Germany). Production of recombinant LAPTc in *E*. *coli* BL21(DE3)pLysS was induced for 20 h at 25°C with 1 mM IPTG and yielded soluble and active enzyme. The protein was purified in two steps by Immobilized Metal Cation Affinity Chromatography (IMAC) and Gel Filtration. For IMAC the nickel matrix was equilibrated with five column volumes (CV) of cold binding buffer (50 mM Tris–HCl pH 8.0, 300 mM NaCl). After loading 10 mL of the protein extract, the column was washed with the same buffer until the absorbance at 280 nm stabilized at the baseline. Next, the column was washed with 5 CV of cold washing buffer [50 mM Tris–HCl pH 8.0, 300 mM NaCl, 50 mM imidazole (Sigma, USA)]. Finally, the protein was eluted with 5 CV of cold elution buffer (50 mM Tris–HCl pH 8.0, 300 mM NaCl, 400 mM imidazole). The eluates were desalted by gel filtration chromatography, using a NAP-10 column (Sephadex G-25 Medium; Sigma, EUA) to eliminate the imidazole.

### Aminopeptidase activity assay by RapidFire-MS

This assay was previously described [16]. The assay was performed in 384-well clear F-bottom polypropylene plates with a final reaction volume of 15 μL. The reaction mixture contained 7.5 μL LAPTc in 50 mM Tris-HCl, pH 7.5, 0.005% NP-40 and 7.5 μL LSTVIVR peptide substrate (Cambridge Research Biochemicals, Billingham, UK) in the same buffer was added to start the reaction. The reaction was performed at room temperature for 40 min and then stopped with 85 μL 1% formic acid containing 0.15 mg/mL STVIVR* internal standard (Cambridge Research Biochemicals, Billingham, UK).

### High-throughput single point screening for LAPTc inhibitors by RapidFire-MS

Single point screening of a library of 3329 compounds was performed in 384-well clear plates (Greiner 781101) at room temperature. Test compounds (45 nL in DMSO) were transferred to assay plates by an ECHO 550 acoustic dispenser (Labcyte). LAPTc was tested at 3 nM with 150 μM (approximately the value of the apparent Michaelis-Menten constant -appK_M_- [16]) LSTVIVR peptide substrate in a 40 min reaction. Before addition of substrate, LAPTc was preincubated with 45 nL compounds or DMSO for 15 min. Compounds, dissolved in DMSO, were tested at 30 μM. Controls without compound (the same volume of DMSO, 0% inhibitory effect) and without enzyme and compound (100% inhibitory effect) were prepared. The experiment was performed without replicates. The remaining experimental conditions were maintained as previously described [16]. Data were processed and analysed through ActivityBase XE (IDBS). The selection criterion for hits in this single point screen was percent inhibition larger than the mean plus three standard deviations. All primary screening data are available in ChEMBL (see Data Availability statement).

### Dose-response studies for LAPTc inhibition by RapidFire-MS

To generate half-maximum inhibitory concentration (IC_50_) data for LAPTc, 10-point dose-response curves with 100 μM as highest concentration and 1:2 dilution in DMSO (0.195–100 μM range) were prepared in 384 well plates. All other experimental conditions were as described above. All IC_50_ curve fitting was performed by four-parameter logistic dose-response curve fit using ActivityBase XE (IDBS). At least three replicates were generated for each hit compound.

### Mode of inhibition studies by RapidFire-MS

Five concentrations of the LSTVIVR peptide substrate (100–1600 μM range) were tested in the presence of 3 nM LAPTc for 40 min. For each substrate concentration, compound 4 was tested at 0, 0.1 and 0.2 μM. For each substrate and compound 4 concentration, a negative control without enzyme was used. Before addition of substrate, LAPTc was preincubated with compound 4 for 15 min. Other experimental conditions were as previously described [16]. K_i_ was calculated by fitting the Morrison equation to the experimental data. Binding energy (ΔGb) was calculated from K_i_ according to the equation ΔG = -RTlnK_i_.

### Dose-response studies for human LAP3 inhibition by RapidFire-MS

Human LAP3 enzyme (Assay Genie, Ireland) was tested at 150 nM with 600 μM (~1 appK_M_) LSTVIVR peptide substrate and 1 mM ZnCl_2_ in a 180 min reaction. All other experimental conditions were as described above for dose-response assays with LAPTc.

### Culture of Vero cells

Vero cells (African green monkey kidney cells, ECCAC 84113001) were maintained in culture as previously described [33]. Briefly, these cells were maintained at 37°C and 5% CO_2_ in MEM supplemented with 10% FCS, sub-culturing every 2–3 days at a ratio of 1:5 after 5 min treatment with Trypsin-EDTA (Gibco).

### *T*. *cruzi in vitro* culture

*T*. *cruzi* parasite, TcI strain Silvio X10/7 subclone A1 [34] was maintained in culture as previously described [33]. Parasites were maintained as amastigotes by passaging on a weekly basis in Vero cells. Culture maintenance infections were carried out at an MOI (multiplicity of infection) of 1.5.

### *T*. *cruzi* intracellular assay

This assay was performed as described [35] with the only modification that treatment was 96 h instead of 72 h. Briefly, Vero cells were infected overnight with tissue culture derived *T*. *cruzi* trypomastigotes in T225 tissue culture flasks (MOI 5). Any remaining free trypomastigotes were washed away with serum free MEM and the infected Vero cells were harvested by trypsinisation. Compounds (250 nL in DMSO) were dispensed using LabCyte ECHO (Beckman Coulter Life Sciences, USA) into each well of Corning black flat bottomed 384-well plates (Corning, USA). Ten-point potency curves were generated (1:3 dilutions in DMSO), with a highest concentration of 50 μM. The infected Vero cells were then plated into the plates containing the compounds, at 4,000 cells per well in MEM media with 1% FCS. After 96 h incubation at 37°C in presence of 5% CO_2_, the plates were fixed with 4% formaldehyde for 20 min at room temperature and stained with 5 μg/mL Hoechst 33342. The plates were imaged on a Perkin Elmer Operetta high-content imaging system using a 20× objective. Images were analyzed using the Columbus system (Perkin Elmer). The algorithm first identified the Vero nuclei followed by demarcation of the cytoplasm and identification of intracellular amastigotes. This algorithm reported percent infected Vero cell and total number of Vero cells. All potency determinations were carried out in at least three independent replicates and are reported as pEC_50_ +/- standard deviation. pEC_50_ = -log(EC_50_[M]).

### Cytotoxicity assay on human HepG2 cells

The assay was performed as reported [36]. Briefly, HepG2 cells were incubated for 72 h with compounds, followed by a resazurin-based read-out (fluorescence, excitation 528 nm and emission 590 nm) with a plate reader.

### Docking studies

The 3D structure of compound 4 was generated with the graphical drawing interface Avogadro version 1.2 [37]. All rotatable torsion angles of compound 4 were defined as flexible. There is only one LAPTc structure in the PDB database (PDB: 5NTG [15]). One of the Mn^2+^ atoms (absent in the original structure) was manually added from superimposition of structures of LAPTc and the acidic M17 LAP from *T*. *brucei*, *Tb*LAP-A (PDB: 5NSM [15]). AutoDock Tools v1.5.6 (ADT) [38] was used to prepare the protein and ligand (compound 4) for simulations, and UCSF Chimera v1.14 [39] to analyze the output.

All hydrogen atoms were added to the molecules, Gasteiger charges were calculated, non-polar hydrogens were eliminated, and the AD4 atom-type assigned to each atom, following Verma *et al*. [40]. A grid box of 26 Å × 26 Å × 26 Å and centred at the coordinates of X: 58.144, Y: 85.476 and Z: 90.384 was used to cover the entire enzyme active site. Docking parameters were kept at default values, except the following: energy_range = 4 kcal, num_modes = 20.

Docking simulations were performed with Autodock Vina [41] for a total of 100 models, and conducted five times. LAPTc residues were considered rigid. Different conformers of the compound 4-LAPTc complex were grouped using as criterion a root mean square deviation (RMSD) value ≤ 2 Å. In each group, the conformation with the lowest free energy of binding, according to AutoDock Vina scoring function, was selected as the representative conformation.

### Optimization of the compound 4-LAPTc complex

To optimize the conformation of the compound 4:LAPTc complex, molecular dynamics simulations were performed, using NAMD v2.12 [42] and the force field CHARMM36 [43,44]. As the starting structure, the best representative conformation of the compound 4:LAPTc complex, according to the binding energy value obtained from molecular docking, was used. The parameters for compound 4 were obtained using the fftk (force field tool kit) plug-in [45] implemented in VMD (Visualizer Molecular Dynamics) [46]. For non-protein components of receptor, i.e. Mn^2+^, parameters were obtained from Won [47]. Vacuum molecular dynamics simulations were performed, using a NVT ensemble, making flexible only amino acid residues located at less than 10 Å from the ligand. Temperature was set to 310 K and was controlled with the Langevin thermostat [48]. Time step was 2 fs and simulations were run for 1 ns, after an energetic minimization of 1000 steps. Data were processed in VMD [46].

### Stability analysis of the compound 4-LAPTc complex

As starting structure, the final structure from the vacuum molecular dynamics simulations, selected as representative of the compound 4-LAPTc complex, was used. The *in silico* complex was solvated and charge neutralized by adding Na^+^ and Cl^-^ at 0.05 M, using the solvate and autoionize plug-ins, respectively, from VMD [46]. A cubic solvation box of 20 Å^3^ was generated from the complex surface to the borderline of the box, using the explicit solvent model TIP3 [49]. Simulations were carried out under periodic boundary conditions. Molecular dynamics simulations were performed using NAMD v2.12 [42], and the CHARMM36 force field [43,44]. An NPT ensemble was used, with temperature at 310 K and pressure at 1 bar, controlled by the Langevin thermostat and barostat [50], respectively. All systems were subjected to 1000 minimization steps before runs. The simulations were performed during 100 ns with intervals of 2 fs.

### Analysis of interactions involved in complex stabilization

Hydrophobic interactions and hydrogen bonds of the final positions in compound 4-LAPTc complex, selected by molecular dynamics simulations, were analyzed using LigPLot+ v2.1 [51]. Analysis of hydrogen bonds throughout the entire trajectory was performed using the hydrogen bond plug-in implemented in VMD [46]. The 3D structure of the representative binding mode for the compound 4-LAPTc complex was visualised with Chimera v1.14 [39].

### Energy calculation using LIE-D

Binding energy calculations for the compound 4-LAPTc complex was performed using LIE-D methodology [52]. Briefly, this method considers the electrostatic (polar) and van der Waals (non-polar) energies to calculate the binding free energies in protein-ligand complexes.

## Results

### High-throughput screen for LAPTc inhibitors

**Fig 2:**
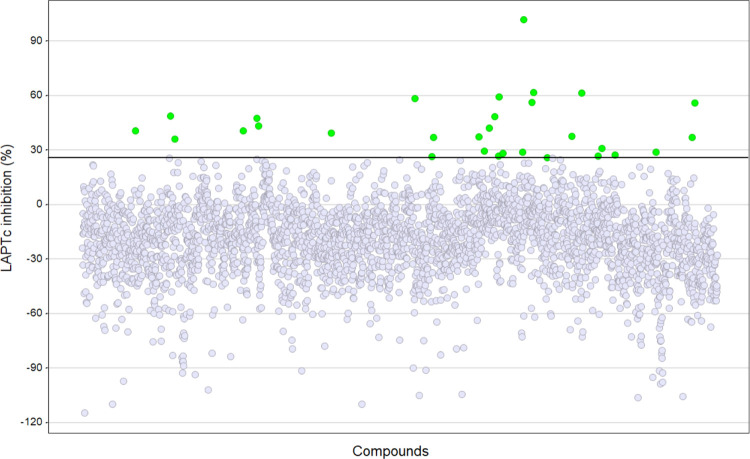
Primary screen. Percent inhibition of LAPTc for each compound in the screen. Compounds were tested in single replicate at 30 μM using the RapidFire-MS enzymatic assay. Hit selection threshold is indicated by the black line, hits are coloured green.

A selected and targeted set of 3,329 compounds that harbour common protease inhibitor motifs was screened at 30 μM against LAPTc using a previously standardized RapidFire-MS method [16]. For hit selection a threshold of 25.8 percent inhibition was selected (mean response plus three standard deviations) (Fig 2), resulting in 30 putative active compounds or hits (S1 File). Screen performance was good, with a Z’ robustness factor of 0.76 ± 0.05 and a signal-to-noise ratio of 64 ± 7. The hits were progressed to 10-point dose-response studies.

### Dose-response characterisation of hits by RapidFire-MS

**Fig 3:**
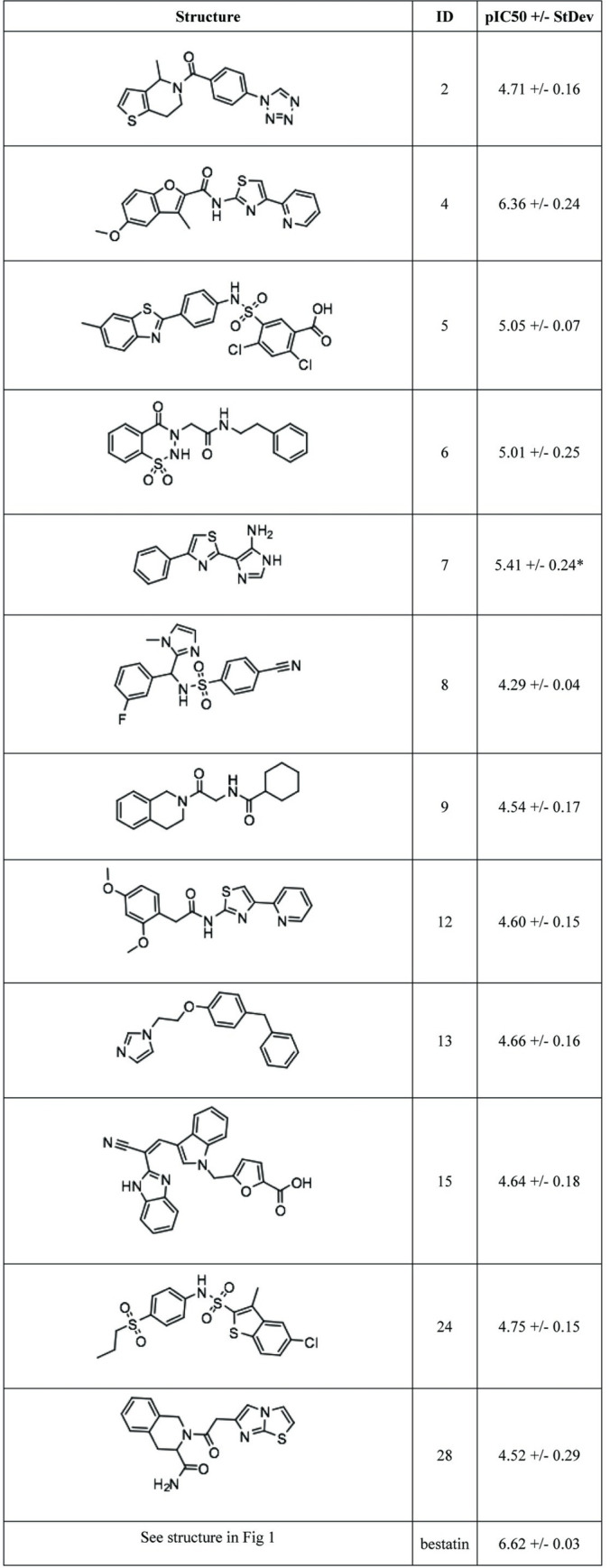
Potency determination for LAPTc screening hits. Compound structures and pIC_50_ values against LAPTc as determined by dose-response studies with the RapidFire-MS enzymatic assay. Data from four independent replicates. * = one replicate was deemed inactive (maximum effect < 50%), average and standard deviation are for the three active replicates.

To confirm the initial hits and assess their potency, single replicate dose-response studies against LAPTc were performed using the RapidFire-MS method. As compounds 3 and 16 were not available, only 28 compounds were tested. Bestatin, a known LAPTc inhibitor, was included as a reference. Dose-response profiling showed that 11 compounds were inactive, five had a maximum effect below 50% (indicating partial inhibition of the enzyme) and 12 compounds had promising IC_50_ profiles (S2 File). For these 12 compounds we generated a further three dose-response curve replicates and the resulting average potency is shown in Fig 3 (see S2 File for data for all individual replicates). Potencies were generally in the low micromolar range. Compound 4 was the most potent, with a pIC_50_ of 6.36 (IC_50_ 0.44 μM).

### *In vitro* activity against intracellular *T*. *cruzi* amastigotes and cytotoxicity

**Fig 4:**
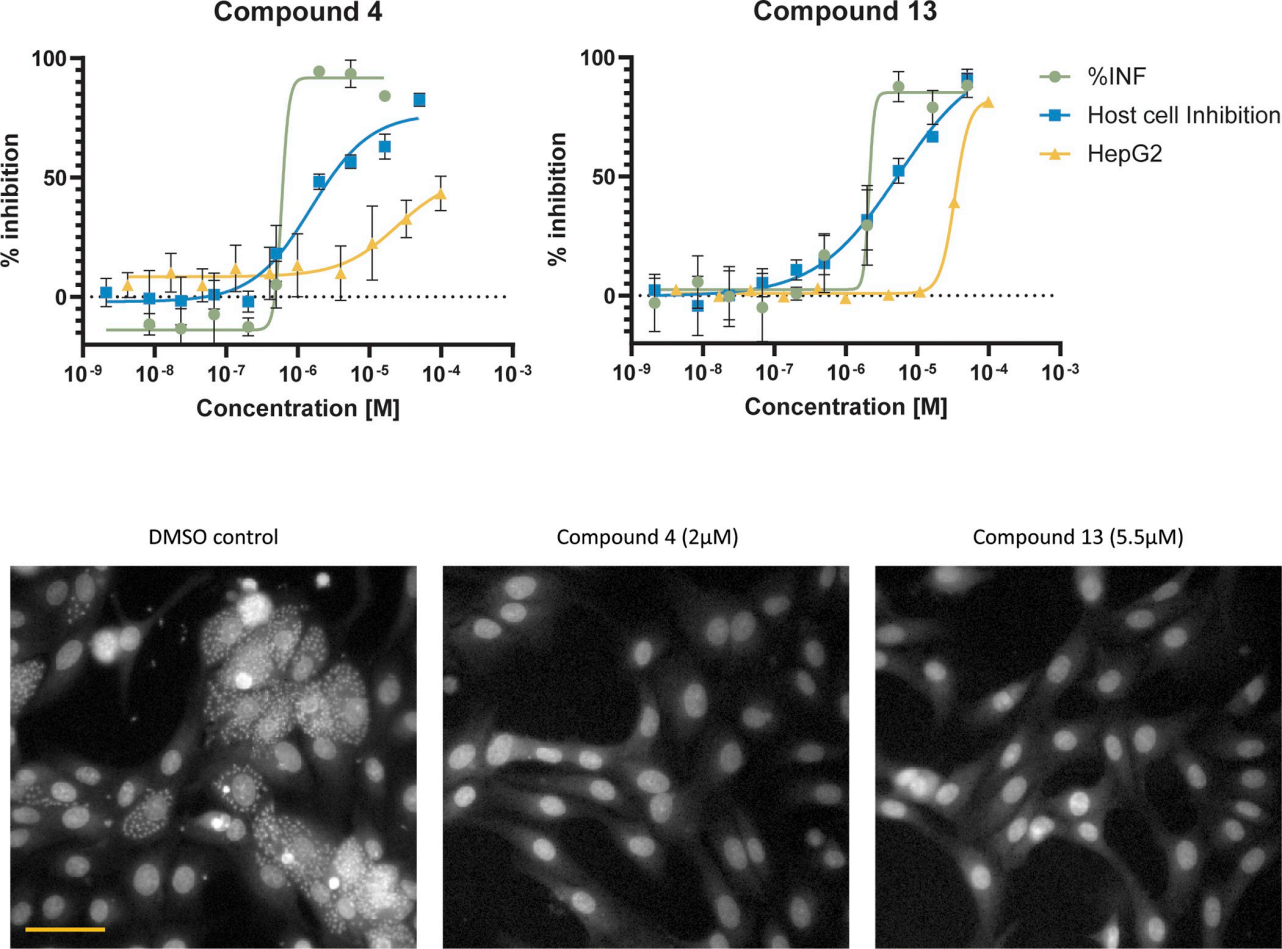
Activity against intracellular *T*. *cruzi* amastigotes for compounds 4 and 13. Top panels: Dose response curves for compounds 4 and 13. Green and blue curves are from the high-content imaging assay, green = percent of host cells infected with parasites (%INF; normalised to 100% effect (16 μM nifurtimox) and 0% effect controls (DMSO)) and blue = percent inhibition of Vero host cells (normalised to 100% effect (no cells) and 0% effect control (DMSO). Data points are average of four independent replicates, standard deviation is shown as error bars. Yellow curve is from the HepG2 assay (see methods). Data points are average of two independent replicates, standard deviation is shown as error bars. Bottom panels: Fluorescence images from the high-content screening assay. Hoechst 33342 was used to stain parasite and host cell DNA. Large structures represent host cell nuclei, small dots are parasite nuclei. Scale bar is 50 μm.

Intracellular amastigotes are the most relevant form of *T*. *cruzi* for human disease as this is the form that resides within host cells. To assess if the hit compounds possess antiparasitic effects, we determined the potency of the 12 hits against intracellular *T*. *cruzi* amastigotes using *in vitro* high-content imaging. As part of the analysis, cytotoxicity against the infected Vero host cells was also measured. While most compounds were either inactive or lacked selectivity, compounds 4 and 13 showed promising potency against intracellular amastigotes (pEC_50_ 6.17 (EC_50_ 0.7 μM) and 5.67 (EC_50_ 2 μM), respectively) and exhibited some selectivity over host cell toxicity (Fig 4, Table 1, and S3 File). Fig 4 shows potency curves for both compounds and representative images from the potency assay to illustrate the antiparasitic effects.

**Table 1:** Potency against intracellular *T*. *cruzi* amastigotes, VERO host cells and HepG2 cells.

Compound	pEC_50_ (*T*. *cruzi*) +/- StDev	pCC_50_ (Vero) +/- StDev	Selectivity index (fold window Tc/Vero)	pCC_50_ (HepG2)
2	<4.3	<4.3		<4
4	6.17 +/- 0.07	5.65 +/- 0.29	3.3	<4
5	<4.3	4.48 +/- 0.07		<4
6	<4.3	<4.3		<4
7	*4.41 +/- 0.12	4.99 +/- 0.21		<4
8	<4.3	<4.3		<4
9	<4.3	<4.3		<4
12	5.15 +/- 0.03	6*		<4
13	5.67 +/- 0.10	5.30 +/- 0.09	2.3	4.36
15	<4.3	4.42 +/- 0.09*		<4
24	4.83 +/- 0.20	4.94 +/- 0.07		<4
28	<4.3	<4.3		<4

*T*. *cruzi* potency determinations were carried out in four independent experiments, HepG2 potency determinations in two independent experiments.

* indicates that compound was inactive in some replicates, in this case the average is calculated only for the active replicates.

The potency of compounds 4 and 13 is comparable to that reported previously in this assay for currently used antichagasic drugs, i.e. nifurtimox and benznidazole (pEC_50_ 6.1 (EC_50_ 0.8 μM) and 5.7 (EC_50_ 2 μM), respectively) [33]. To further investigate potential host cell toxicity, we tested the effect of the compounds on uninfected HepG2 cells, a commonly used mammalian cytotoxicity model. Interestingly, all 12 hit compounds showed no or significantly less toxicity against HepG2 cells compared to infected Vero cells (Fig 4, Table 1 and S4 File). The effect of compound 4 was too small to allow determination of a half-maximal cytotoxic concentration (CC_50_) and compound 13 displayed a pCC_50_ of 4.36 (CC_50_ 43 μM). Selectivity windows based on the HepG2 data were thus substantially larger at >166-fold and 20-fold respectively, with the caveat that some toxicity was seen for compound 4 at 33 μM and 100 μM.

The structures of compounds 4 and 13 are shown in Table 1. Both are low-molecular-weight compounds, with hydrophobic and voluminous functional groups, as is expected for inhibitors of a M17 LAP. Both structures are nitroheterocycles; compound 4 with four rings (two of them condensed) and compound 13 with three rings. Taking into account that compound 4 showed the best results at enzymatic and cellular level, it was selected for further characterisation.

### Determination of mode of inhibition for compound 4

**Fig 5:**
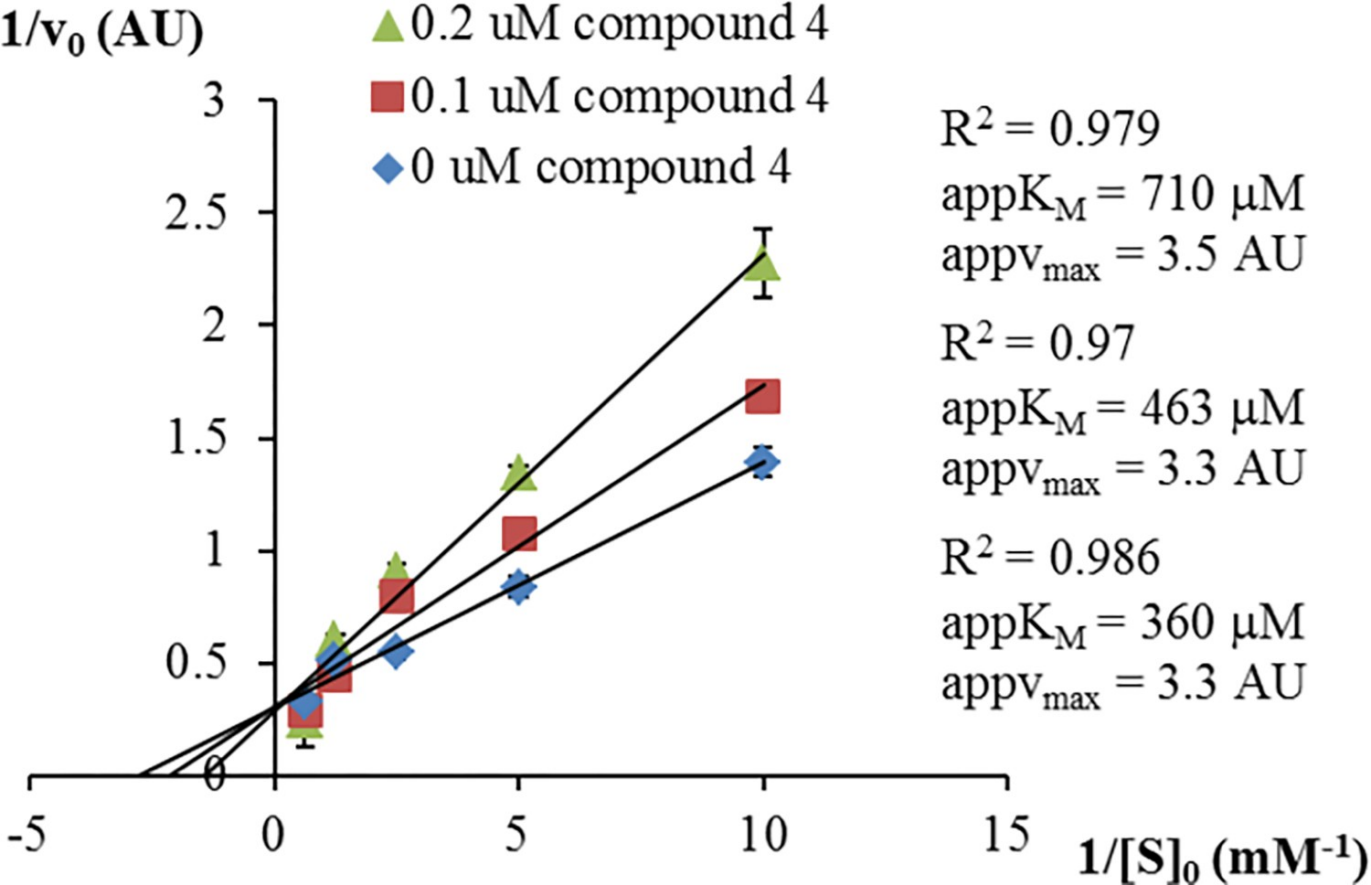
Determination of the mode of inhibition of LAPTc by compound 4, with respect to the LSTVIVR peptide substrate using the RapidFire-MS enzymatic assay. Double reciprocal Lineweaver-Burk plots are shown. Determination coefficient values (R^2^) for the linear fittings are shown, and data are presented as means ± standard deviations (n = 3). v_0_: initial velocity. AU: arbitrary units. appK_M_: apparent K_M_. appv_max_: apparent maximal velocity. [S]_0_: initial substrate concentration.

Mode of inhibition studies for LAPTc enzyme were performed with compound 4 using the RapidFire-MS method described above. Double-reciprocal Lineweaver-Burk analysis demonstrated that compound 4 is a competitive inhibitor with respect to the substrate peptide LSTVIVR (Fig 5), with K_i_ of 0.27 μM, as determined by fitting the Morrison equation to the experimental data.

### Selectivity of compound 4 over human LAP3

To determine if compound 4 inhibition of LAPTc was selective, we determined the IC_50_ for human LAP3, the closest human homologue of LAPTc. Concentrations up to 100 μM exhibited no inhibition of LAP3. Hence with an IC_50_ for human LAP3 is > 100 μM the selectivity index of compound 4 for LAPTc is > 500. By contrast, bestatin has a pIC_50_ against LAP3 of 6.60 +/- 0.05 (IC_50_ = 0.25 μM) and therefore has no selectivity between LAPTc and human LAP3.

### Modelling of LAPTc inhibition by compound 4 by molecular docking *in silico*

**Fig 6:**
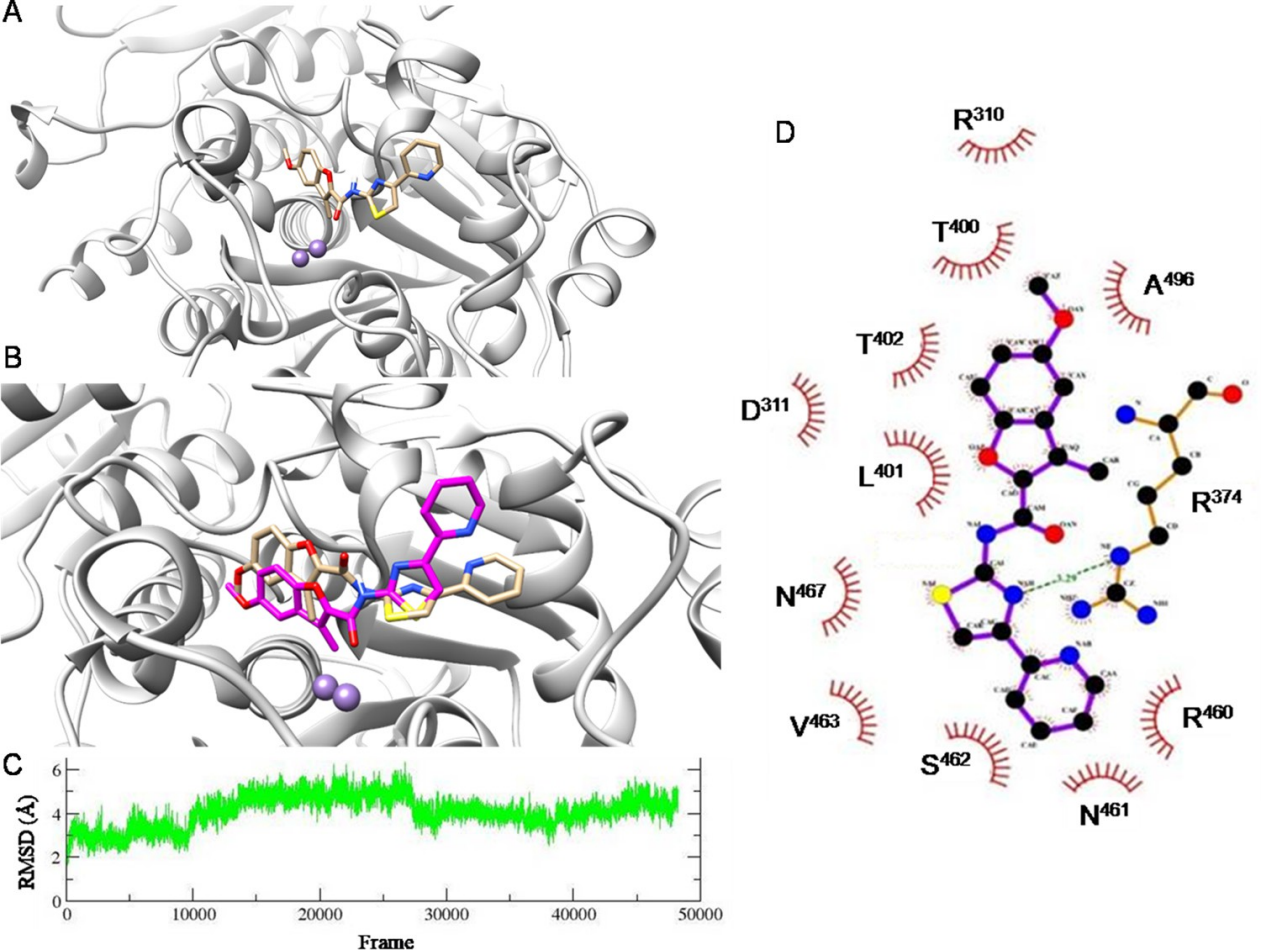
Modelling of LAPTc inhibition by compound 4 by molecular docking *in silico* and molecular dynamic simulations. (A) Structure of the selected conformer of the compound 4-LAPTc complex, according to its binding energy value, obtained by molecular docking *in silico* (AutoDock Vina software). (B) Energy optimization of the compound 4-LAPTc complex by vacuum molecular dynamics simulations. Compound 4 (in sticks) in its initial conformation (beige) and after 1 ns of vacuum molecular dynamics simulations (magenta) is shown. The enzyme active site is shown in cartoon (light grey), with the two Mn^2+^ atoms represented as purple spheres. (C) Temporal course of RMSD variation in the solvated molecular dynamics simulations for the compound 4-LAPTc complex. 500 frames = 1 ns. (D) 2D representation of predicted stabilizing interactions between compound 4 and LAPTc after 100 ns of solvated molecular dynamics simulations. Compound 4 and R^374^ residue (carbons represented in black) are shown in balls and sticks model. Hydrophobic interactions between amino acid residues and compound 4 are shown with red curve lines, and the hydrogen bond with a green dashed line. In (A), (B) and (D) nitrogen atoms are represented in blue, oxygens in red, sulfurs in yellow and hydrogen in white.

LAPTc inhibition by compound 4 was modeled by molecular docking *in silico*. For this, the only LAPTc 3D structure available in PDB (PDB: 5NTG [15]) was used to build a functional dimer. Rotatable bonds of the ligand were considered flexible and LAPTc amino acid side chains were taken as rigid. Seven conformers of the compound 4-LAPTc complex were obtained, and we selected the conformation with the best binding energy value (-8.9 kcal/mol) for energy optimization (Fig 6A). This value is consistent with the experimental value (-8.95 kcal/mol). Compound 4 binds the LAPTc active site in the substrate binding site (Fig 6A). Therefore, the molecular docking result is also consistent with the competitive inhibition mode experimentally determined.

### Energy optimization of the compound 4-LAPTc complex

To optimize the conformation and minimize the energy, modelling of the compound 4-LAPTc complex was continued by vacuum molecular dynamics simulations for 1 ns. Fig 6B shows the conformational and positional changes of compound 4 relative to its initial position. The inhibitor hardly changed its position in the active site. Slight variations up to 2 Ǻ were observed, mainly associated with the rotation of the pyridyl ring. Based on these coordinates, stability analysis was carried out for 100 ns in a solvated system.

### Stability analysis of the compound 4-LAPTc complex

Solvated molecular dynamics simulations were performed for 100 ns to assess the stability of the compound 4-LAPTc complex. RMSD variation analysis for the compound 4-LAPTc complex in a 100 ns simulation demonstrated that the inhibitor conformation remained stable over time with very few fluctuations (Fig 6C). Compound 4 showed RMSD values ​​below 4 Ǻ. The binding energy calculated for the complex by the LIE-D [52] was -8.9 kcal/mol, again consistent with the experimental value (-8.95 kcal/mol). Stability is a consequence of hydrophobic interactions between compound 4 and the side chains of R^310^, D^311^, R^374^, T^400^, L^401^, T^402^, R^460^, N^461^, S^462^, V^463^, N^467^ and A^496^ residues (Fig 6D). Only one hydrogen bond interaction was predicted, between R^374^ and the compound 4 thiazole ring. Notably, inhibitor coordination of metal cations in the enzyme active site is not predicted.

## Discussion

Interest in metalo-aminopeptidases has increased recently, mainly promoted by involvement in essential physiological processes and relevance to pathogenesis [12]. In parasites, aminopeptidases are involved in infection of mammalian cells, proliferation, differentiation, defence, dissemination through host tissues and others [53]. Specifically, M17 metalo-aminopeptidases have been identified as therapeutic targets; for example PfA-M17 from *P*. *falciparum* [22].

LAPTc is active in the three main parasite life cycle stages: epimastigote, trypomastigote and amastigote [14]. Taking into account the absence of the biosynthetic pathway for leucine [14,15], *T*. *cruzi* must acquire this amino acid through recycling and/or from the environment [54]. Intracellular amastigotes are the key life-cycle stage for pathogenesis in Chagas disease [3]. Inside the host cell amastigotes are exposed to low free amino acid concentrations, and hence parasite LAP activity could play a major role in leucine supply through hydrolysis of exogenous and endogenous peptides [14]. Therefore, LAPTc is an attractive target for identifying inhibitors with antichagasic activity. Specific LAPTc inhibition/inactivation may also clarify its roles in parasite survival/development in the human host.

*In situ* inhibition of LAPTc by bestatin in epimastigotes suggests that LAPTc can be inhibited by low-molecular-weight bestatin-like compounds in intact parasites [20]. Identification of new inhibitors is particularly valuable in context of Chagas disease as a worldwide health problem [2] and the lack of new drugs in the development pipeline.

To address this we performed a high-throughput screen for LAPTc inhibition by a methodology based on the identification and quantification of the enzymatic reaction product by mass spectrometry. While RapidFire-MS has been used in high-throughput screens to identify inhibitors for other enzymes, such as sphingosine kinase [55], arginase II [56], LRRK2 kinase [57], demethylase-1 [58], monoacylglycerol acyltransferase [59], histone lysine demethylases [60] and acetyl-coenzyme A carboxylase [61], this is the first report of applying this method to identify inhibitors of an aminopeptidase. The assay performed well with a Z’ robustness coefficient of 0.76 ± 0.05 and signal-to-noise ratio of 64 ± 7, indicating suitability for high-throughput screening.

The confirmed LAPTc inhibitors exhibit structures consistent with typical M17 LAP inhibitors. All are low-molecular-weight compounds with hydrophobic and voluminous functional groups, able to establish hydrophobic interactions with the enzyme active site pockets. These structural requirements are common for M17 LAP inhibitors reported by other authors [21,22,28–31]. The most potent inhibitor found here, compound 4 (pIC_50_ 6.36, IC_50_ 0.44 μM) has a terminal pyridyl group linked to a thiazole ring (Table 1). At the other end, compound 4 has two condensed rings (furan and phenyl) and a methoxy group. Interestingly, compound 12, which shares the thiazole-pyridyl side of compound 4 but has an *m*-dimethoxyphenyl group at the other end (Table 1) is 40-fold less active against LAPTc providing some initial structure-activity relationship insight and supporting the importance of a double ring system in compound 4 for high-affinity binding. It is also interesting that the four most potent inhibitors (compounds 4, 7, 28 and 5; Table 1) share the thiazole ring. These novel LAPTc inhibitors complement the small cohort of previously described LAPTc inhibitors (including bestatin [14], arphamenine A [16] and the bestatin-derivative peptidomimetic KBE009 [27]; Fig 1), and provide new opportunities to explore this mechanism of action as a therapeutic target for Chagas disease as well as tool compounds to investigate basic biology. Compound 4 is highlighted as a candidate for further progression and the only molecule with a submicromolar IC_50_ for LAPTc inhibition.

When tested against intracellular amastigotes, compounds 4 and 13 demonstrate anti-parasitic activity. However, the selectivity window against Vero host cells was narrow (Fig 4), but both compounds showed less cytotoxicity against HepG2 cells, a common cell line used for cell health experiments. The poor selectivity for compound 4 in infected Vero cells is not driven by inhibition of the closest human homologue of LAPTc as no inhibition of LAP3 was detected. It will be important to characterise the mechanism driving cellular toxicity, but it is encouraging that less toxicity was seen in HepG2 cells compared to infected Vero cells. A caveat is that compound exposure for uninfected HepG2 cells was 24 hours shorter than in infected Vero cells. In addition, the toxicity in Vero cells may be compounded by the presence of dying parasites. Thus, future work should explore effects of incubation time on cellular toxicity, in HepG2 cells and infected versus uninfected Vero cells. Additional studies are also required to demonstrate that the compounds are active across multiple strains from different DTUs, and to confirm that the antiparasitic activity of compounds 4 and 13 is mainly driven through inhibition of LAPTc.

With respect to selectivity between LAPTc and human LAP3, optimised conditions were used for each enzyme with substrate concentration ~1 appK_M_, enzyme concentration at 3 and 150 nM respectively, and over 40 and 180 minutes respectively [16]. Ultimately, confirmation of the physiological level of selectivity should be obtained in relevant cellular models through measurement of inhibition of substrate cleavage.

Compound 4 is a competitive inhibitor of LAPTc, with K_i_ 0.27 μM. The compound has a good ligand efficiency (LE) of 0.33 (LE = -RT ln K_i_ / number of non-hydrogen atoms) and shows high selectivity over the closest human homologue. Together, this makes compound 4 an excellent starting point for a LAPTc drug discovery campaign.

An *in silico* binding model for the compound 4-LAPTc complex was generated, and provides structural guidance for medicinal chemistry development of compound 4. The model is consistent with compound 4 being a competitive inhibitor and the predicted binding energy (-8.9 kcal/mol) is in line with the experimental value (-8.95 kcal/mol), providing validation for the model. The main predicted interactions between compound 4 and LAPTc are hydrophobic, with hydrogen bonds less abundant (Fig 6D). This is consistent with the hydrophobicity of the active site.

These enzymes have an S1 sub-site formed by hydrophobic and negative residues, and an S1’ pocket mainly formed by hydrophobic and positive amino acids [62]. Although S1 and S1’ sub-sites have not been described for trypanosomatid M17 LAPs, they have been described for PfA-M17. The narrow hydrophobic S1 pocket of PfA-M17 is formed by M^392^, M^396^, F^398^, T^486^, G^489^, L^492^ and F^583^ residues [63]. Hydrophobic residues A^460^ and I^547^ are key residues of the S1’ cavity [64].

Bestatin binds to the PfA-M17 active site through hydrophobic interactions. The benzyl group in bestatin’s P1 position (Fig 1) interacts in the PfA-M17 S1 sub-site with the hydrophobic amino acids M^392^, M^396^, F^398^, G^489^ and A^577^. The isobutyl side chain in bestatin’s P1’ position (Fig 1) contacts the N^457^ and I^547^ residues [63]. In addition, bestatin coordinates the divalent metal cations of PfA-M17 [63] and *Tb*LAP-A [15]. For compound 4, interaction with LAPTc’s metal cations is not predicted. A similar hydrophobicity-driven interaction has also been modelled for the bestatin-based peptidomimetic KBE009 [27] (Fig 1), with hydrophobic interactions between KBE009 and LAPTc residues K^300^, F^304^, D^370^, T^402^, G^403^, A^496^ and F^497^ (numbering differs in one residue). Two of these residues also participate in the predicted interaction with compound 4 (T^402^ and A^496^).

A binding-mode based on hydrophobic interactions rather than metal cation coordination could be favourable for inhibition selectivity [65], as binding would not be directed by the conserved and strong coordination with the metal cations, but by many, weaker individual interactions (mainly van der Waals interactions, with to a lesser extent ionic interactions and hydrogen bonds). Such interactions could exploit the structural differences between the active sites of the parasite and human aminopeptidases, potentially allowing potent and specific inhibition of LAPTc. Importantly, LAPTc and human LAP3 have differences in their hydrophilicity/hydrophobicity at the entrance to their catalytic pockets and LAP3 also has a relatively hydrophilic area close to the catalytic site. This is thought to drive the selectivity of KBE009, a relatively hydrophobic compound, for LAPTc over LAP3 [27] (Fig 1). A similar effect may explain the selectivity we observe for compound 4, which is also relatively hydrophobic.

## Conclusions

We identified multiple novel LAPTc inhibitors through mass spectrometry-based high-throughput screening. Compound 4, in particular, is of interest as a potent, selective and competitive LAPTc inhibitor, with a submicromolar K_i_. We propose a binding mode for this compound, based on docking and molecular dynamics evidence, that is in excellent agreement with experimental data. Compound 4 exhibits *in vitro* antichagasic activity against *T*. *cruzi* amastigotes, and while toxicity against the host cells needs to be explored further, the compound provides a valuable starting point for Chagas disease drug discovery going forward.

## Supporting information

S1 File RF LAPTc hits.xlsx: Structures, compounds identifier and percent inhibition from primary screen for the 30 hits. (XLSX)

S2 File LAPTc pIC50 DRC.xlsx: Potency data for hits in RapidFire-MS assay. pIC_50_ = -LOG(IC_50_[M]). Experiment column indicates if potency was obtained from initial potency confirmation or subsequent replicate generation experiments. Hit confirmation column indicates if hits from primary single concentration screen confirmed in the potency experiment.
(XLSX)

S3 File Tcruzi pEC50 DRC.xlsx: Potency data for intracellular parasites (T. cruzi) and host cells (Vero). pEC_50_ = -LOG(EC_50_[M]).
(XLSX)

S4 File HepG2 pEC50 DRC.xlsx: Potency data against HepG2 cells. pEC_50_ = -LOG(EC_50_[M]).
(XLSX)
